# Research on the New Model of Data-Driven Teaching Decision-Making for University Minority Language Majors

**DOI:** 10.3389/fpsyg.2022.901256

**Published:** 2022-06-16

**Authors:** Mian Ru

**Affiliations:** College of Foreign Languages, Huaiyin Normal University, Huai’an, China

**Keywords:** data-driven, minority language major, teaching decision-making, precision, result analysis mode

## Abstract

This article expounds on the urgency of the reform of the training mode of university minority language majors, elaborates on the data-driven teaching decision-making mode, and explores the implementation strategy of teaching decision-making driven by data. Multimodal teaching behavior data and refined process analysis method promote precision and personalized teaching, change the traditional thick line result analysis mode, and accelerate the development of minority language major teaching.

## Introduction

The “Belt and Road” strategy has established bilateral cooperation between China and 65 countries across Asia, Europe, and Africa, and has enhanced the economic construction and cultural exchanges [1]. There are a total of 60 official languages in these countries (Hui and Yalan, 2016), and 18 of these official languages are not currently offered as courses in Chinese universities [3]. The “Belt and Road” strategy has made the gap in language talents more and more obvious, especially in the case of minority languages (Wei, 2010). Minority languages are the name for non-universal languages. According to the regulations of the United Nations, languages other than Chinese, English, French, Russian, Spanish, and Arabic are minority languages (Yu and Hongdan, 2022). The “Belt and Road” strategy brings challenges and opportunities to minority language majors, which need to inject fresh blood into their modes of training and talent output structure. We need to integrate data into the teaching and decision-making and explore a new path for the construction of minority language majors.

With the advent of big data, data has become an important factor in production (Mickinsey Global Institute, 2011). It subverts the traditional experience imitation teaching mode of the minority language major, moves toward a data-driven teaching mode, and promotes innovation in teaching. In recent years, governments at home and abroad have emphasized the important role of data-driven decisions in teaching. In October 2020, the blue book on the development of big data in China’s basic education (2020–2021) defined the overall development and trend of big data in the field of domestic basic education. Let data become an important support and leading force to promote domestic precision teaching reform, and let data-driven become a path and method to promote the modernization of education governance [7]. In May 2020, the University of California adopted a data-driven approach to support the empirical research of education policy. Some European countries use student performance data to evaluate teachers’ teaching (Organisation for Economic Co-operation and Development, 2021). Data-driven teaching decision-making refers to the process of using teaching behavior data to provide decision-making for teaching and organizing the data into an information flow. We refine the information flow according to different teaching needs, make the data have operable attributes, and carry out scientific teaching activities under the support of data (Mandinach, 2012). Data-driven curriculum teaching decision-making theory has become the theoretical basis of teaching reform (Datnow et al., 2013). From the perspective of data motivation, the evaluation and examination data are used to test students’ satisfaction with teaching.

However, many researchers rarely apply data-driven teaching to minority language majors, which is subject to the unclear principle and framework of data-driven teaching decision-making (Herman and Gribbons, 2001). In view of this situation, this paper attempts to study the connotation and characteristics of data-driven teaching, analyze the urgency of the reform of the training mode of minority language majors, and explore the data-driven teaching decision-making process. On this basis, this paper puts forward the design scheme of data-driven teaching decision-making, which provides direction and guidance for teachers’ curriculum teaching development.

The organization of the paper is arranged as follows: The second chapter discusses the urgency of minority language major training mode reform, the third chapter discusses the data-driven teaching decision-making mode for minority language major courses, the fourth chapter discusses the design method of data-driven teaching decision-making, the fifth chapter discusses the implementation strategies of data-driven teaching decision-making, and the sixth chapter summarizes the paper.

## The Urgency of Minority Language Major Training Mode Reform

### The Demand for Minority Language Talents

The “Belt and Road” strategy, proposed in 2013, and China joining the WTO in 2001, have transitioned China’s economy toward an export-oriented model. An export-oriented economy is an economic type in which countries or regions take the international market demand as their guidance, and export expansion is the central driver of economic development (Shuai, 2014). Under this circumstance, universities urgently need to cultivate minority language talents to meet the language needs of countries along the “Belt and Road” strategic line. In recent years, many universities have expanded the enrollment of minority language majors. Some universities plan to set up the “Belt and Road” language research center, and gradually establish national language courses of countries along the “Belt and Road” strategic line. Some foreign universities are also making efforts to train minority language talents [13]. By 2021, there are more than 510 Japanese major courses, more than 120 Korean majors, more than 110 German majors, and more than 20 Italian majors in Chinese universities. Many universities are speeding up the construction of minority language majors to fill the dearth of minority language talents, but they also face the problems of poor development of minority language disciplines and low teaching quality. This puts forward requirements for the quality of minority language talents. It is necessary to integrate big data into teaching decision-making, and change the traditional “scholastic” minority language major teaching mode to an “accurate and applied” teaching model.

### The Employment Demand for a Minority Language Major

The “Belt and Road” strategy has accelerated the development speed of minority language majors, changed the employment areas and fields of minority language talents, and promoted innovation in minority language major teaching modes. In the past, the employment area for minority language talents was mainly concentrated in China’s second-line cities or registered residence. Minority language students were mainly engaged in foreign affairs, education, tourism, and foreign trade. In recent years, countries along the “Belt and Road” line, such as those in Africa and Latin America, have been chosen by many minority language talents. Some changes have taken place in the job market. In the traditional employment field, the demand for foreign language translation is reduced, and the employment is diversified, involving logistics, the Internet, and service industries, which requires students to have high comprehensive abilities. Emerging cross-industries based on minority language majors are becoming more and more popular, such as cross-border e-commerce, foreign trade, and cross-border logistics. These works combine foreign languages with other majors to meet the requirements of emerging industries. Small and medium-sized enterprises are particularly prominent. They can quickly understand the changes in market demands. More small and medium-size enterprises are developing their market along the “Belt and Road” countries. The demand for jobs combined with minority languages and finance, marketing, logistics, and other professional services has increased significantly. This requires that the university should not only pay attention to the study of minority languages, but also combine different majors. To innovate teaching methods in the training modes, we need to apply data-driven teaching decision-making to the teaching of minority language majors, and let students have a structured knowledge structure.

### The Need for the Minority Language Major’s Training Mode Reform

The training model of minority language students commonly uses the method of “minority language + professional major,” takes language as the main learning purpose, and integrates professional knowledge such as countries’ human geography, economy, and culture into language learning. This model can enable students to have the ability to listen, speaking, reading, and writing, master the essential laws of language theory in that country’s language, and have certain professional knowledge. But such students are not competent for jobs in professional fields. Some minority language majors adopt the mode of a “double degree,” taking a minority language major as their first major and another major as a second major. After completing two majors, students can obtain a “double degree.” This model can enable students to obtain greater academic advantages in employment, but due to the limitation of training time, the depth of knowledge gained from the second major is relatively shallow. The models of “minority language + professional major” and “double degrees” take the ability to listen, speaking, reading, and writing as students’ language skills. The cultivation of professional knowledgeability is easy despise and cannot give full play to the advantages of a minority language in the field of professional knowledge. At present, university minority language majors in Chinese universities are mainly Japanese, German and Korean. The teaching time lags behind that of English, and there is a lack of a mature minority language training system. This requires reform and innovation in the training mode and talent output structure of minority language majors, to break with the previous teaching and training mode. We need to integrate information technology into the training of minority language majors, and apply data-driven teaching decision-making to this model.

## Data-Driven Teaching Decision-Making Modes for Minority Language Majors

### Data-Driven Teaching and Its Main Features

With the rapid development of information technology, big data has been integrated into every field of modern life. Many researchers are also actively exploring the path of data-driven teaching decision-making (Wenmei et al., 2021; Xiaoqing and Shijin, 2021). Cloud computing, ubiquitous network, virtual teaching and other technologies extend the learning space, break the traditional physical teaching space, and run through online and offline teaching. The teaching model has changed from traditional breakpoint teaching to online and offline continuous teaching, and the teaching content is displayed in multimodal forms through multiple types of teaching media (such as mobile terminals and virtual devices). The data-driven model can use teaching media to store and transmit the teaching behavior data of teachers and students formed in multiple environments and use artificial intelligence technology to visually analyze and process the data. This can sort out the relationship between various types of data, mine the internal value of data, conduct multivariate analysis, provide an auxiliary reference for teaching decision-making, improve teachers’ precision teaching, guide students’ precision learning, and form a positive feedback teaching mechanism. It can improve the quality of teaching plans, teaching interaction, and teaching reflection, form a data-driven teaching mode and provide two-way support for teachers and students. In the teaching process of minority language majors, data-driven teaching decision-making needs to collect all kinds of behavior data of teachers and students, extract features of structured and unstructured data, build user portraits or user models of each student, and form personalized teaching decision-making schemes.

Data-driven teaching decision-making takes data as the support, innovates the links of design and implementation in teaching, changes the traditional experience teaching mode, and raises scientificity and accuracy of teaching. Multiple terminals (such as intelligent wearable devices, and mobile devices) enrich the teaching behavior data, extract the data of the whole learning process (learning progress, learning habits, learning hobbies), refine the data granularity, and enhance the data accuracy. By mining a large amount of teaching behavior data, we can effectively grasp the students’ knowledge acquisition status, group commonalities, and individual blind spots, find the hidden group laws and personality characteristics, improve the accuracy of user portraits, and promote the link of fine teaching design, organization, guidance, and intervention.

### The Reform of Minority Language Major’s Teaching Decision-Making

**FIGURE 1:**
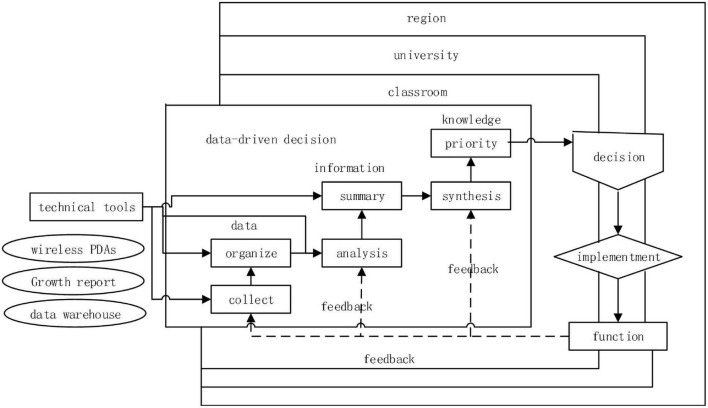
Data-driven decision model of technical support.

In the mode of data-driven teaching decision-making, the teaching behavior data of teachers and students are collected and stored based on the online and offline teaching modes. The teaching content is displayed in the teaching media (such as mobile devices, and virtual teaching platforms) in the form of video, sound, pictures, and text. Teaching behavior data is automatically saved in the media terminal. The value of teaching behavior data is extracted through data mining technology to provide data support for teaching decision-making. Mandinach first put forward the theoretical framework of data-driven teaching decision-making, envisaged that technical tools can be used to support, enable and promote data-driven decision-making, and tried to assist teachers in using the data-driven teaching decision-making model through technology development (Mandinach et al., 2006). The proposed model is shown in Figure 1. The added value of using technology in data-driven decision-making will become more and more obvious, which can realize the functions of data mining and meaning extraction. Data-driven practice has changed the mode of teaching decision-making and formed a new model of teaching for minority language majors, which is mainly reflected in the changes in decision-making thinking and decision-making ideas, decision-making subjects, and decision-making subjects’ data literacy.

#### The Reform of Decision-Making Thinking and Concept

Data-driven teaching is formed on the big data, which uses big data technology to collect massive and heterogeneous teaching behavior data. Through mining the internal relationship of data, we can master the learning state of students in each stage, and adjust teaching contents and methods in real-time. The traditional teaching decision-making can be described as phenomenon observation–problem identification–cause analysis–scheme demonstration–decision implementation. At present, the concept of English teaching is relatively mature, and the teaching research is relatively extensive, which can be used for reference for minority language majors. But this mode cannot fully adapt to minority language majors. This is because each language has a relative theory. Minority language majors’ teaching is carried out relatively late in China, and the combination of textbook compilation and curriculum is not perfect. It is necessary to integrate data-driven teaching decisions into the minority language major, enrich the teaching theory, and deal with the in-depth and extension problems existing in the curriculum. The teacher puts forward a clear curriculum teaching goal, subdivides teaching tasks, collects teaching behavior data in the teaching process, excavates the logic and regularity of data, and then feedback to the teaching plan. This method can adjust the teaching content, and make up for the defect that the combination of teaching materials and courses for minority language majors is not high.

Data-driven teaching decision-making subverts traditional decision-making and changes the way of decision-making thinking. Through data mining and technical analysis of phenomena, problems, and other events, we can reveal the internal relationship between data. This reflects the relevance of multiple teaching behavior data sets, explores the laws and development trends of multiple teaching behavior data sets, and effectively integrates the data of teachers, students, and teaching behavior.

#### The Reform of Decision Concerned Subject

In the traditional teaching decision-making, the language learning of minority language students mostly starts at University. Freshmen and sophomores learn the basic knowledge of the language, and sophomores and seniors learn the cultural background of the language. Compared with English majors, the foundation is poor. The learning of theoretical courses is more difficult, and there is a lack of reasonable language teaching methods. This makes teachers unable to effectively obtain the teaching behavior data of each student and make accurate personalized teaching decisions. In the data-driven teaching mode, teachers can effectively obtain the teaching behavior data. The analysis based on the data can accurately grasp the needs and learning status of each student, adjust the teaching plan in time, and realize the personalized teaching scheme and intervention guidance of teaching students according to their aptitude. It can describe in detail the learning process and cognitive progress of each student in a minority language major, and formulate reasonable teaching objectives, teaching plans, and teaching plans for each student. This changes the traditional teaching decision-making, which aims at the problems of the whole class or most of the students. This way can turn the focus from the whole to the individual, and achieve the goal of paying attention to all students in the real sense.

#### The Reform of Decision-Makers’ Data Literacy

The differences and dynamics of the teaching experience of minority language teachers contain their own learning experience, scientific research experience, values, and other background factors, all with a certain personal coefficient (Polanyi, 1974). It will affect the effectiveness and rationality of teaching decision-making, which is a behavior of testing “real impact” through “trial and error” (Qing and Yishu, 2016). It is easy for teachers to form abnormal and narrow teaching philosophies. The introduction of data-driven provides a way to solve teachers’ over-dependence on their own personal teaching experience. It uses data to present the relationship logic and allows the algorithm to represent the characteristics of the data. The unprocessed teaching behavior data is a group of numbers without any value. After data processing, it will reflect the value of knowledge, assist teachers in teaching decision-making, correct the teaching thinking formed by relying on teaching experience and form a comprehensive cooperation mode.

Teachers are the main body of data-driven teaching decision-making. Their data literacy can promote the operation of data—information—action knowledge—decision-making practice, and will also affect the implementation and completion of teaching decision-making. Teachers’ data literacy is an important link in the development of data-driven teaching decision-making. It is urgent to formulate teachers’ data literacy standards, establish training systems at all levels, and cultivate the formation of teachers’ data literacy (Yonghe et al., 2012). Integrating big data technology and artificial intelligence technology into teaching decision-making can establish an effective training evaluation and intervention mechanism. Integrating the data literacy training platform into higher education can build a data-driven teaching and training system. Integrating the data literacy recognition standard into the evaluation system can formulate the data literacy evaluation mechanism. Through these methods, teachers can continuously accumulate data wisdom in the process of practice, absorb the nutrition of educational reform, and have the ability to analyze, interpret, exchange, discover, and apply data.

## Design Method of Data-Driven Teaching Decision-Making

**FIGURE 2:**
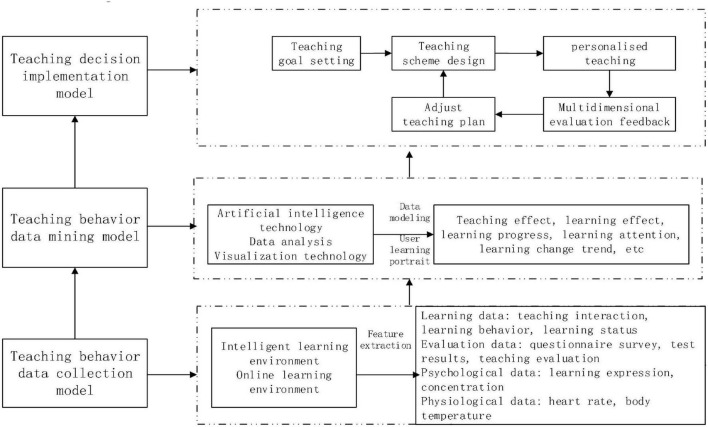
Teaching decision-making scheme of a minority language major.

Some studies have shown that unreliable and inaccurate data can affect the application of teaching decision-making. Teachers know little about cognitive strategies to transform data into available information and practice, which is the main reason that data application is not paid attention to in front-line teaching at present (Tuytens and Devos, 2011). Information technology turns the teaching environment from a physical space (such as classroom and library) to an online virtual space and turns the data form from its traditional state as text to video, voice, and other states. This makes the complex of data calculation and processing and increases the difficulty of data-driven teaching decision-making and teaching optimization in minority language majors. Establishing an effective data-driven teaching decision-making method is a problem. This paper designs a teaching decision-making scheme for data-driven minority language majors from three aspects: teaching behavior data collection, teaching behavior data mining, and teaching decision-making execution, as shown in Figure 2.

### Teaching Behavior Data Collection Model

Information and intelligent technology change the teaching environment from a single simple form to a multi-modal complex form, and also makes the data model from a single text state to a variety of voice, image, video, and other states. This requires the construction of a multi-source data collection mode integrating online and offline, which can collect learning data, evaluation data, psychological data, and physiological data. This method can ensure the integrity of teaching behavior data and make the data mining results more scientific.

In the past, teaching behavior data was mainly extracted from online learning environments, which is easy to collect for the characteristics of openness and interaction. However, the diversity of teaching environments (such as family, library, and network) makes the data form multi-source, which brings differences to data analysis methods. Online collection of teaching behavior data mainly uses web crawlers, web logs, online learning platforms, and background databases to extract the data of teaching interactive communication, learning behavior, and media operation. An intelligent learning environment can be used for offline collection of teaching behavior data, which can automatically record the learning process, identify students’ psychological and physiological characteristics, evaluate learning results, and perceive learning situations. The data of students’ psychological and physiological characteristics are the data of students’ learning expression, concentration, heart rate, body temperature, and EEG, which is obtained by using cameras, face recognition technology, and intelligent wearable devices. In the intelligent learning environment, smart wearable devices and mobile devices can obtain the behavior data of students’ learning interests, learning participation, classroom interaction, knowledge acquisition, and other links in different environments and physical conditions. This can record the interactive data between teachers and students in multiple dimensions, integrate the learning behavior data in multiple scenes, and improve the structured teaching behavior data. These data are preprocessed by cleaning, sorting, classification, statistics, and classification, and the data is characterized by feature extraction. The mapping relationship between teaching behavior data and eigenvalues is constructed to facilitate later data mining.

### Teaching Behavior Data Mining Model

Teaching behavior data mining is using artificial intelligence, data analysis, visualization, and other technologies to model behavior data, and mine the state results of teachers and students (such as teaching effect, learning effect, learning progress, learning attention, and learning habits) for later teaching decision-making.

The traditional manual data analysis methods cannot effectively analyze the teaching behavior data in the face of a complex teaching environment. It is necessary to integrate artificial intelligence, quantitative analysis, visualization, and other technologies into data-driven teaching decision-making to weaken the influence of teachers’ subjective factors on data analysis. Deep learning using feature extraction is the development direction of automatic data analysis in the future. It promotes the combination of domain knowledge and teaching behavior data model to form a process analysis model with specific properties. Behavioral data modeling and learning user portraits are the core of data-driven teaching decision-making. It is necessary to determine the relevant definition criteria in the teaching field. According to the teaching environment and teaching needs, the teaching behavior data mining model could select the initial model suitable, take the data eigenvalues formed in the teaching behavior data collection stage as the input value, and form an implantable and popularized behavior recognition model through data training. In the later stage, the robustness of the model is enhanced by adding constraints, the iterative convergence and stability of data training are improved, and the results of the teaching behavior state are excavated (such as teaching effect, learning effect, learning progress, learning attention, learning change trend, learning habit, learning emotion influencing factors).

The teaching behavior data mining model can help teachers extend their cognition to students from the surface behavior state to the implicit state, from the previous shallow teaching mode of topic style description to the deep teaching mode of internal analysis of teaching effect, learning effect, learning emotion and learning motivation.

### Teaching Decision Implementation Model

The teaching decision-making is mainly to deal with the external application of the analysis results in the teaching behavior data, including behavior analysis, online decision-making, and prediction analysis, to provide a personalized learning environment and implement personalized teaching.

Based on data mining, the teaching decision-making implementation model feeds back the behavior data model results to the teaching decision-making, uses the internal relationship of teaching behavior data to design the teaching scheme, forms personalized teaching, and effectively improves the quality of teaching. Through the feedback of students’ teaching behavior data in the course, adjust the teaching scheme (such as teaching interaction mode, and interaction time) to match the teaching needs of different types of students’ learning progress. In the teaching decision-making implementation model, multi-dimensional evaluation feedback plays an important role. It is necessary to improve the effectiveness and diversity of forms of evaluation, effectively serve the design of the teaching scheme, and form a circular teaching operation of “design—execution—feedback— adjustment.”

The accuracy and rationality of teaching decisions are supported by data mining, and the subjective experience of traditional teaching is turned into the objective reasoning of “Internet + teaching.” Based on the teaching behavior data, the teaching decision model could mine the teaching state results through the behavior data model, and combine the results with the specific teaching environment to form a reasonable teaching decision. This can carry out multi-dimensional evaluation and feedback on the results of teaching-learning, form a complete set of closed-loop operations, and effectively think about the process of teaching-learning.

## Implementation Strategies of Data-Driven Teaching Decision-Making

Multimodal teaching behavior data and refined process analysis methods promote the accurate and personalized teaching of minority language majors, change the traditional thick line result analysis mode, and promote the development of a data-driven teaching mode. This mode has begun to be used by some educators in teaching and scientific research, but its development power and influence are not enough. There are still some problems, such as coarse particles of teaching behavior data collection and incomplete process analysis. It has not formed an integrated analysis process of data-driven teaching decision-making from the perspective of the whole process. In view of this situation, this paper analyzes the implementation strategy of data-driven teaching behavior data from the aspects of process behavior data, multimodal feature extraction, and the application of teaching decision-making methods.

### Perfect Process Behavior Data

An intelligent learning platform is an important support for the operation of a data-driven teaching mode. We need to establish a multi-source collection platform for teaching behavior data of minority language majors, integrate an intelligent learning environment and online learning environment into the platform, improve the extraction method of process behavior data, and realize the fine perception of the teaching process. This can turn the traditional teaching paradigm based on experience or observation in minority language majors into a teaching model based on process behavior data. Through the natural collection of process behavior data and the seamless connection of multi-source data on the intelligent learning platform, we can deeply mine the continuous teaching interaction data of the dynamic time axis, and apply the web crawler and log analysis to the non-verbal behavior data to improve the fine granularity of teaching behavior data collection of minority language major.

### Optimize Multimodal Feature Extraction

Multimodal feature extraction technology mainly includes statistical analysis, data mining, quantitative analysis, discourse analysis, and information visualization. These technologies are used to refine the original data and complete cleaning, sorting, statistics, and classification. Statistical analysis is a universal statistical analysis of the interaction between teachers and students. Data mining is to find the hidden relationship in the teaching behavior data. Using the relationship between recognition and various achievements can build a multi-dimensional data array. Quantitative analysis is to analyze the relationship between students’ learning state and physiological signs. Discourse analysis is to analyze the relationship between students’ discourse and communication text in the process of online learning. Information visualization is to analyze the relationship between teachers’ or students’ learning process and results, and present teachers’ teaching situation and data mining results in a visual way.

The accuracy of teaching behavior data’s feature extraction will affect data analysis and data mining. The multimodal feature extraction method can mine the whole process of teaching behavior data through the complementarity and correlation of multi-source data, and analyze the quantitative indicators such as internal emotion, teaching emotion, and cognitive acquisition.

### Apply the Teaching Decision-Making Methods

The application of the teaching decision-making method is to test the effect of data feature extraction and behavior data model. It is to effectively complete the teaching objectives and contents, implement the teaching scheme, solve the problems existing in the process of teaching design, and improve the scientificity of the teaching model. Different teaching environments will form a diversified dynamic process, which requires effective analysis of teaching behavior data to avoid the teaching mode dominated by teachers’ subjective experience. The data-driven teaching method with data as the core and leading will become the mainstream model. Teachers also need to pay attention to the risk of data rights brought by data cave, combine data-driven with a practical teaching environment, infiltrate theory into practice and build an integrated analysis process. In this process, data-driven teaching transmits its energy and value in the technology layer, method layer, and application layer. The technical layer is the basis of teaching behavior data analysis, including statistical analysis, data mining, quantitative analysis, discourse analysis, information visualization, and other technologies. The method layer is the means of data analysis, including behavior data modeling, and behavior user portrait. The application layer is the effective output of the technology layer and method layer, including behavioral analysis, and prediction analysis.

## Conclusion

Relying on artificial intelligence, data analysis, visualization and other technologies, data-driven teaching decision-making of minority language majors will lead to the transformation of traditional teaching mode in decision-making thinking, attention to subject, and data literacy. From the aspects of teaching behavior data collection, teaching behavior data mining, and teaching decision execution, this paper designs a data-driven teaching decision-making scheme for minority language majors, ensure the integrity of teaching behavior data, make the data mining results more scientific and accurate and build an accurate and personalized teaching system.

## Data Availability Statement

The original contributions presented in this study are included in the article/supplementary material, further inquiries can be directed to the corresponding author.

## Author Contributions

MR was responsible for the construction of thesis framework, writing, and modification.

## Conflict of Interest

The author declares that the research was conducted in the absence of any commercial or financial relationships that could be construed as a potential conflict of interest.

## Publisher’s Note

All claims expressed in this article are solely those of the authors and do not necessarily represent those of their affiliated organizations, or those of the publisher, the editors and the reviewers. Any product that may be evaluated in this article, or claim that may be made by its manufacturer, is not guaranteed or endorsed by the publisher.
